# Supplementary material for: Changes in Food Consumption, BMI, and Body Composition in Youth in the US during the COVID-19 Pandemic
Source: Int J Environ Res Public Health. 2023 Sep 21;20(18):6796. doi: 10.3390/ijerph20186796 (PMC10531233; doi:10.3390/ijerph20186796)
Supplement: Supplementary file 1 [file ijerph-20-06796-s001.zip › ijerph-2564139-supplementary.pdf]

International Journal of
Environmental Research and Public Health

MDPI

Supplementary Materials

Table S1. Within-Group Differences in Total Energy Intake, BMI, BMIz, and Adiposity.

	No Pandemic Group				Pandemic Group			
	Baseline M, SD	Difference[1] M, SD	t	*p*	Baseline M, SD	Difference[1] M, SD	t	*p*
**BMI**	20.76, 4.69	+ 2.24, 2.44	-6.96	<.001*	22.57, 6.18	+ 1.66, 3.50	-3.20	.003*
**BMIz**	0.45, 0.94	+ 0.02, 0.49	-1.00	.318	0.74, 1.10	- 0.15, 0.56	2.37	.021*
**Adiposity (%)**	53.64, 10.39	+ 0.69, 6.24	-1.26	.208	56.66, 10.10	- 1.17, 5.83	1.99	.050*
**Energy Intake (kCal)**	949.82, 424.31	+ 77.83, 407.70	-2.85	.005*	1046.47, 405.97	+ 174.43, 611.60	-3.35	.001*

[1]Difference is the change between baseline and 3-year visit values calculated as 3-year visit minus baseline. *Significance assuming α=.05.

Table S2. Differences in the Change of Total Energy Intake Before and During the Pandemic.

	Total Energy Intake (kcal) Difference Score			
	Fully Adjusted		Partially Adjusted	
**Covariates**	F	*p*	F	*p*
Model	1.57	0.13	1.96	0.15
Group	0.41	0.52	0.62	0.43
Age	2.20	0.14	--	--
Sex	2.02	0.16	--	--
Race	0.01	0.94	--	--
Ethnicity	0.50	0.48	--	--
Height	1.33	0.25	--	--
Time Between Visits	<0.01	1.00	--	--
Lean Mass	0.92	0.34	--	--
Adiposity	6.18	0.02*	2.89	0.09

Group was operationalized as No Pandemic (0) and Pandemic (1). *Significance assuming α=.05.

Table S3. BMI, BMIz, and Adiposity Differences at the 3-Year Visit Before and During the Pandemic.

	BMI			
	Fully Adjusted		Partially Adjusted	
**Covariates**	F	*p*	F	*p*
Model	5.37	<.001*	12.10	<.001*
Group	0.58	0.45	0.66	0.42
Age	21.62	<.001*	22.88	<.001*
Sex	0.55	0.46	--	--
Race	1.64	0.20	--	--
Ethnicity	0.02	0.90	--	--
	**BMIz**			
	**Fully Adjusted**		**Partially Adjusted**	
**Covariates**	F	*p*	F	*p*
Model	1.36	0.26	N/A	N/A
Group	0.08	0.78		
Race	3.00	0.09		
Ethnicity	0.26	0.62		

Height	2.11	0.15		
		Adiposity		
	**Fully Adjusted**		**Partially Adjusted**	
**Covariates**	F	*p*	F	*p*
Model	5.05	<.001*	15.10	<.001*
Group	0.05	0.83	0.03	0.86
Age	0.50	0.48	--	--
Sex	19.62	<.001*	30.11	<.001*
Race	0.06	0.81	--	--
Ethnicity	0.23	0.63	--	--
Height	0.52	0.47	--	--

Group was operationalized as No Pandemic (0) and Pandemic (1). *Significance assuming α=.05.

Table S4. Change in Percent Calories from Protein Consumed with Covariates.

	**Fully Adjusted Model**	
	**F**	***p***
Model	1.18	0.32
**Group**	**0.79**	**0.38**
Age	0.15	0.70
Sex assigned at Birth	0.71	0.40
Height	0.46	0.50
Race	0.08	0.79
Ethnicity	2.91	0.09
Time Between Visits	<0.01	0.99
Lean Mass	0.15	0.70
Adiposity	6.20	0.01*

Group was operationalized as No Pandemic (0) and Pandemic (1). *Significance assuming α=.05.

Table S5. Change in Percent Calories from Fat Consumed with Covariates.

	**Fully Adjusted Model**	
	**F**	***p***
Model	1.43	0.19
**Group**	**0.12**	**0.74**
Age	0.01	0.91
Sex assigned at Birth	0.10	0.75
Height	0.05	0.83
Race	0.79	0.38
Ethnicity	4.53	0.04*
Time Between Visits	0.03	0.86
Lean Mass	<0.01	0.96
Adiposity	6.59	0.01*

Group was operationalized as No Pandemic (0) and Pandemic (1). *Significance assuming α=.05.

Table S6. Change in Percent Calories from Carbohydrates Consumed with Covariates.

	**Fully Adjusted Model**	
	**F**	***p***
Model	1.06	0.40
**Group**	**1.45**	**0.23**
Age	0.24	0.62
Sex assigned at Birth	<0.01	0.98
Height	1.36	0.25
Race	0.18	0.67

Ethnicity	0.44	0.51
Time Between Visits	1.38	0.24
Lean Mass	2.37	0.13
Adiposity	0.47	0.50

Group was operationalized as No Pandemic (0) and Pandemic (1).

Table S7. Change in Percent Calories from Fiber Consumed with Covariates.

	Fully Adjusted Model	
	F	*p*
Model	0.73	0.68
**Group**	**0.07**	**0.79**
Age	0.53	0.47
Sex assigned at Birth	0.01	0.92
Height	0.97	0.33
Race	0.04	0.85
Ethnicity	0.76	0.38
Time Between Visits	0.02	0.90
Lean Mass	0.70	0.40
Adiposity	2.47	0.12

Group was operationalized as No Pandemic (0) and Pandemic (1).

Table S8. Change in Percent Calories from Sugar Consumed with Covariates.

	Fully Adjusted Model	
	**F**	***p***
Model	1.29	0.25
**Group**	**0.25**	**0.62**
Age	0.13	0.72
Sex assigned at Birth	2.00	0.16
Height	1.30	0.26
Race	0.09	0.77
Ethnicity	0.53	0.47
Time Between Visits	1.35	0.25
Lean Mass	3.10	0.08
Adiposity	<0.01	0.97

Group was operationalized as No Pandemic (0) and Pandemic (1).

Table S9. Change in Percent Calories from Saturated Fatty Acids Consumed with Covariates.

	Fully Adjusted Model	
	**F**	***p***
Model	1.24	0.28
**Group**	**0.05**	**0.83**
Age	0.07	0.79
Sex assigned at Birth	<0.01	0.97
Height	0.13	0.72
Race	0.19	0.67
Ethnicity	3.77	0.07
Time Between Visits	0.99	0.32
Lean Mass	0.08	0.78
Adiposity	3.91	0.05

Group was operationalized as No Pandemic (0) and Pandemic (1).

Table S10. Change in Percent Calories from Monounsaturated Fatty Acids Consumed with Covariates.

	Fully Adjusted Model	
	**F**	***p***
Model	1.66	0.12
**Group**	**1.02**	**0.31**
Age	0.01	0.91
Sex assigned at Birth	0.02	0.90
Height	0.05	0.82
Race	2.55	0.11
Ethnicity	2.56	0.11
Time Between Visits	0.25	0.62
Lean Mass	0.03	0.87
Adiposity	5.45	0.02*

Group was operationalized as No Pandemic (0) and Pandemic (1). *Significance assuming α=.05.
